# Single‐Cell Motility Rapidly Quantifying Heteroresistance in Populations of *Escherichia coli* and *Salmonella typhimurium*

**DOI:** 10.1002/smsc.202100123

**Published:** 2022-03-24

**Authors:** Giampaolo Pitruzzello, Christoph G. Baumann, Steven Johnson, Thomas F. Krauss

**Affiliations:** ^1^ Department of Physics University of York York YO10 5DD UK; ^2^ Department of Biology University of York York YO10 5DD UK; ^3^ Department of Electronic Engineering University of York York YO10 5DD UK

**Keywords:** antimicrobial resistance, bacterial motility, heteroresistance, hydrodynamic trapping, microfluidics

## Abstract

Heterogeneous bacterial populations can display increased resistance to external threats, such as exposure to antibiotics. Despite the mounting clinical evidence supporting the importance of bacterial heterogeneity in acute infections, current antimicrobial susceptibility tests (ASTs) are typically insensitive to cell‐to‐cell differences as they only measure population‐wide averages. Herein, the use of single‐cell motility to address this issue is demonstrated. It is shown for the first time that antibiotic susceptibility detected as a change in single‐cell motility is an excellent proxy for polyclonal and monoclonal heteroresistance. It is also demonstrated that motility and growth are both inhibited by an antibiotic with strikingly similar patterns, thus enabling the quantification of minimum inhibitory concentration (MIC) using a high‐throughput, single‐cell motility assay. The method allows for the detection of heteroresistance in *Escherichia coli* and *Salmonella typhimurium* in 2 h or less and quantifies the MIC of an antibiotic in 1.5 h. The findings emphasize the need for characterizing bacterial heterogeneity, and they highlight the importance of single‐cell bacterial motility in assessing both antibiotic susceptibility and population‐wide heteroresistance.

## Graphical abstract

A key issue in the global fight against anti‐microbial resistance is that heterogeneous bacterial populations can display varied responses to antibiotics. Herein, it is demonstrated that single‐cell bacterial motility is a rapid, quantitative, and high‐throughput reporter of heteroresistance through a microfluidic approach based on single‐cell trapping.

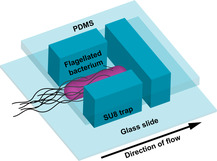

## 1 Introduction

Heterogeneity is present in all bacterial populations,^[^ ^1^ ^]^ driven by the intrinsic stochasticity of the processes involved in gene expression and protein biosynthesis or by the response to an external challenge that triggers phenotypical differentiation.^[^ ^2^, ^3^ ^]^ Importantly, heterogeneity is known to play a crucial role during the exposure of bacterial communities to antibiotics.^[^ ^4^ ^]^ Heterogeneity in response to antimicrobials manifests itself via a variety of phenomena commonly described as heteroresistance, whereby a bacterial population separates into two or more subpopulations that show different levels of sensitivity to an antimicrobial challenge. Unfortunately, the concept of heteroresistance is not always clearly defined in the literature and the term itself has been used rather liberally.^[^ ^5^, ^6^ ^]^

Despite the increasing clinical evidence supporting the importance of population‐wide heterogeneity, heteroresistance is often missed in traditional antimicrobial susceptibility tests (ASTs) because typical ASTs only assess the bacterial community in its entirety and use a single average value to describe a sample.^[^ ^6^, ^7^, ^8^, ^9^ ^]^ For example, Band et al.^[^ ^7^, ^10^ ^]^ recently showed that colistin heteroresistance in *Enterobacter cloacae* and *Klebsiella pneumoniae* went undetected by traditional agar plating and led to a failure of treatment in mice infection models. This failure is of particular concern considering that colistin (a bactericidal antibiotic) is a last‐line defense antibiotic and, even though colistin resistance is typically chromosomally encoded, the emergence of plasmid‐mediated mechanisms for the acquisition of resistance has recently been reported in *Enterobacteriaceae*.^[^ ^11^ ^]^ Moreover, currently available techniques for profiling heteroresistance such as population analysis profile (PAP) and genetic profiling are very time and resource intensive. In addition, such methods might miss low‐occupancy subpopulations because they lack single‐cell sensitivity.^[^ ^6^, ^12^ ^]^

In light of the considerations above, more rapid and high‐throughput methods for assessing heteroresistance are required. In addition, a necessary condition for probing population‐wide heterogeneity is clearly the ability to measure single bacteria, which some of the traditional methods (such as disc diffusion) lack. In this context, the ability of microfluidic devices to spatially isolate individual bacteria and monitor them over time has already enabled rapid high‐throughput analysis.^[^ ^13^, ^14^ ^]^ For example, the microfluidic “mother machine” constrains bacterial growth along narrow channels to allow for the long‐term monitoring of single bacteria while controlling their local environment.^[^ ^15^, ^16^, ^17^, ^18^ ^]^ In the context of heteroresistance, the mother machine has been used to link the existence of persister cells to phenotypical suppression of their growth rate.^[^ ^19^ ^]^ In other examples, droplet microfluidics^[^ ^20^, ^21^ ^]^ and electrorotation^[^ ^22^ ^]^ have been used to study different manifestations of heteroresistance, such as persistence or monoclonal heteroresistance.

Several methods have attempted to use nanomechanical cell vibrations or motility to measure bacterial susceptibility to antibiotics. These include tethering bacteria to an atomic force microscope (AFM) tip and measuring the cantilever deflections over time,^[^ ^23^, ^24^ ^]^ monitoring the electrical voltage drop across a microchannel caused by swimming bacteria,^[^ ^25^ ^]^ or tracking the nanomotion of bacteria tethered on a gold surface using surface plasmon resonance imaging (SPRi).^[^ ^26^ ^]^ These assays used spatially constricted bacteria, where free swimming was prevented, to show that population‐wide changes in nanomechanical vibration and motility do occur on much shorter timescales than traditional bacterial growth assays, so that antibiotic susceptibility and resistance could be detected in less than 1 h.^[^ ^23^, ^25^ ^]^ However, these assays do not retain single‐cell resolution information, providing only a population‐wide average measurement of motion and because of this averaging are unable to directly observe different phenotypes in a population of bacteria.

Here, we harness the advantages of microfluidics to demonstrate that bacterial motility at single‐cell resolution can be used as a reporter of heteroresistance. Motility is a useful bacterial property because it is a fundamental characteristic of many pathogenic and nonpathogenic bacteria and vital to both chemotaxis and the colonization of their preferred environmental niche. Flagellated motility is also a key virulence factor for pathogenic bacteria that colonize the mucosal membranes of the lungs, bladder, and intestine^[^ ^27^ ^]^; it is estimated that up to 80% of urinary tract infections (UTIs) are caused by motile uropathogenic *E. coli* (UPEC).^[^ ^28^ ^]^ In addition, the flagellum itself has an additional direct role in surface adhesion, biofilm formation, secretion of effector molecules, and immunogenicity.^[^ ^29^, ^30^ ^]^ Hence, studying motile bacteria is important for quantifying pathogenicity and is of high relevance in clinical diagnosis.

We use hydrodynamic trapping^[^ ^31^, ^32^, ^33^ ^]^ to capture individual Gram‐negative bacteria, which does not require functionalized surfaces (e.g., with antibodies) and can be easily integrated into microfluidic assays. Our method can profile hundreds of individual bacteria in parallel, allowing population‐wide analysis of their motility distribution. Using this assay, we clearly observe a heterogeneous motility response for both monoclonal and polyclonal bacterial populations to antibiotic challenge and verify the observed heteroresistance profiles against traditional growth assays (i.e., broth microdilution and agar plating). Notably, single‐cell motility signatures map directly to polyclonal and monoclonal resistance and can therefore identify heteroresistance in mixed bacterial populations within 2 h of exposure to bacteriostatic or bactericidal antibiotics.

We also show, for the first time, that antibiotics inhibit bacterial motility and growth with remarkably similar patterns and dose dependence. By exploiting this similarity, we demonstrate that single‐cell motility can also be used as an excellent substitute for population‐level growth assays to quantify the minimum inhibitory concentration (MIC) in a fraction of the time, specifically 1.5 h versus >16 h required by traditional growth‐based techniques.

## 2 Results

**Figure 1:**
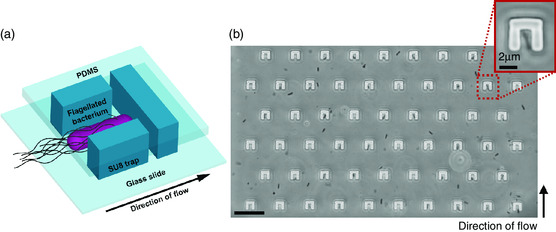
Schematic of the hydrodynamic trapping assay. a) 3D schematic of a single hydrodynamic trap. Traps are fabricated in SU8 on a glass slide and sealed with PDMS microchannels. Bacteria are mechanically retained once they enter a trap. Trapping is monitored with a phase contrast microscope. b) Phase contrast image (top view) of a trapping array with bacteria swimming inside the microfluidic channel. The main scale bar is 25 μm. The inset shows a hydrodynamic trap occupied by a single bacterium.

A 3D schematic of a hydrodynamic trap within the microfluidic channel is shown in **Figure** **1**a, while Figure 1b shows a micrograph of a trapping array taken with a phase contrast microscope. The hydrodynamic trapping array can be fabricated and integrated in a microfluidic device using our previously established procedures^[^ ^31^ ^]^ (see Experimental Section for more details). The traps are sufficiently small to accommodate a single rod‐like bacterium, as shown in Figure 1b and inset. In addition, once a trap is occupied by a bacterium, the fluidic resistance of the trap increases, preventing other bacteria from entering the same trap.^[^ ^33^, ^34^ ^]^

Bacteria were grown in liquid media and injected into the chips using a microfluidic pump (see Experimental Section and Supporting Information for more details). High‐definition videos of bacteria swimming inside the microchannels and entering the traps were recorded and subsequently analyzed to quantify single‐cell motility, as detailed later.

### 2.1 Measurement of Motility

In proof‐of‐concept experiments, we characterized our method by testing bacteria with different motility characteristics. Specifically, we utilized *Escherichia coli* MG1655 and *Salmonella typhimurium* as model motile strains, while *E. coli* BW25113 was used as a nonmotile variant due to its impeded flagellar motility.^[^ ^35^ ^]^ To create additional nonmotile controls (termed “dead” here), the non‐motile *E. coli* and motile *S. typhimurium* strains were exposed to temperatures of 65 °C for 2 h in a block heater. Heat‐induced death was verified by resuspending a small aliquot of these bacteria in fresh medium and incubating overnight at 37 °C. No growth was observed. As a third control, we treated both motile strains with carbonyl cyanide *m*‐chlorophenyl hydrazone (CCCP), which reversibly depolarizes the bacterial inner membrane, thereby preventing rotation of the flagellar motor.^[^ ^36^ ^]^

**Figure 2:**
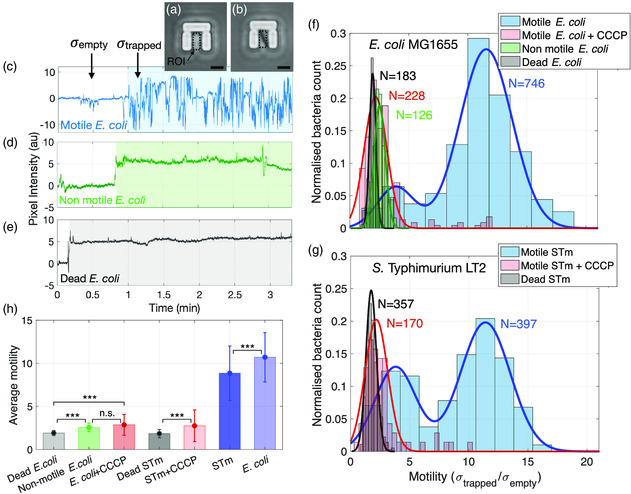
Single‐cell motility of bacterial strains in different conditions. a) Image of an empty and b) occupied trap. The ROI defined for the analysis of pixel intensity is indicated by the dotted boxes. c–e) Typical pixel intensity traces over time for a motile *E. coli* MG1655 (blue), nonmotile *E. coli* BW25113 (green), and dead *E. coli* BW25113 (gray). The shaded regions represent the trapping time. f) Normalized histograms of measured motility values for the four conditions: motile *E. coli* MG1655 (blue), motile *E. coli* MG1655 + 20 μM CCCP (red), nonmotile *E. coli* BW25113 (green), and dead *E. coli* BW25113 (black). Continuous lines are obtained by Gaussian fits of the normalized histogram counts. g) Same conditions as (f), but for *S. Typhimurium* (STm). h) Average motility for all the described conditions. Error bars refer to the standard error over five replicates for the motile populations and two replicates for the other conditions.

Intensity traces are obtained from the recorded videos by calculating the average pixel intensity over time within regions of interest (ROIs) located inside each trap, as shown in **Figure** **2**a,b. Typical traces produced by bacteria caught in single traps are shown in Figure 2c–e. The trapping of a bacterium is indicated by an abrupt change in intensity, while bacterial motion results in signal fluctuations due to the in‐plane and out‐of‐plane movement of the trapped bacterium. Comparing the intensity fluctuations during trapping establishes the link between the intensity traces and the motility, as we have previously demonstrated.^[^ ^31^ ^]^ For example, the motile *E. coli* MG1655 strain produces fluctuations of greater magnitude than that observed for the nonmotile and the dead *E. coli* BW25113 bacteria. We quantified the fluctuations by calculating the standard deviation of the trace produced by a bacterium inside the trap, *σ*_trapped_, during the entire time it was present within the trap (highlighted by the shaded areas in Figure 2c–e). To remove the background noise, *σ*_trapped_ was divided by the standard deviation of the intensity for an empty trap, *σ*_empty_. This unitless ratio, *σ*_trapped_/*σ*_empty_, was used as a proxy for bacterial flagellar motility, as other forms of micro‐scale motion inside the traps are negligible compared with the variance produced by flagellar motor‐driven motion. The validity of these assumptions is further verified by analyzing the distribution of observed motility values in different conditions.

Figure 2f,g shows histograms of the calculated motility distribution for the different bacterial strains and conditions described earlier. The histograms are fitted as Gaussian distributions to retrieve average motilities and the relative spread. We note that trapped motile *E. coli* and *S. typhimurium* cells do not swim homogenously and that their behavior is best described by bimodal Gaussian distributions. The analysis reveals a low‐motility sub‐population centered at motility values of (3.8 ± 2.0) for *E. coli* and (3.7 ± 2.2) for *S. typhimurium*, while the main populations are at (11.6 ± 2.9) and (11.4 ± 2.4) for *E. coli* and *S. typhimurium*, respectively.

In contrast, the nonmotile and dead BW25113 strains produced unimodal distributions with average values of (2.5 ± 0.6) and (1.9 ± 0.4), respectively. The unimodality suggests that all bacteria were motility impaired or dead, respectively, while the difference between the average values shows that it is easily possible to distinguish the viable, nonmotile BW25113 from the same strain following heat inactivation. We also note that the dead strains still produce *σ*_trapped_/*σ*_empty_ ratios >1, which can be mainly ascribed to Brownian diffusion and flow‐induced motion of the dead cells within the traps.

To further elucidate the nature of the measured motion, we considered the CCCP‐treated bacteria and observed unimodal motility distributions with average values of (2.3 ± 1.0) and (2.4 ± 1.1) for *E. coli* and *S. Typhimurium*, respectively (Figure 2f,g). Figure 2h illustrates the average motility values for each of the conditions. The graph clearly shows that the motility of CCCP‐treated motile MG1655 cells (red bars) is not significantly different from that of the nonmotile BW25113 cells (green bar, *p* = 0.098), confirming that flagellated motility is the major contributor to the observed motion prior to CCCP treatment. In contrast, the difference between dead bacteria (black bars) and both the nonmotile BW25133 and CCCP‐treated MG1655 cells is sufficient to be statistically significant (*p* < 0.001). This observation suggests that even when the flagellar motor is inhibited, viable strains engage in other low‐amplitude forms of microscale motion that produce detectable fluctuations above the background noise level.

Similar low‐amplitude motion has been observed in surface‐tethered (where flagellated motility is restricted) bacteria.^[^ ^24^, ^26^, ^37^, ^38^ ^]^ This type of motion has been ascribed to fluctuations in the cell wall present in metabolically active bacteria. It is interesting to note that our hydrodynamic traps can detect the small difference between dead bacteria and live bacteria undergoing low‐amplitude microscale motions not caused by rotation of the flagellar motor. This observation is supported by the fact that both nonmotile and CCCP‐treated cells produce statistically significant higher‐variance signals compared with dead bacteria (see Figure 2h) even though their flagella do not rotate. It is also worth mentioning that, unlike surface‐tethered bacteria, the recorded fluctuations in our assay include both lateral and vertical flagellar‐driven movements, as bacterial motion is not restricted by surface attachment.

A further question that arises from the measured histograms of motility concerns the nature of the observed bimodality for the motile strains. We have explored this aspect by calculating the time‐dependent standard deviation using a sliding window of 5 s length that is moved over the entire duration of the trapping event. This analysis shows that the high‐amplitude microscale motion produced by individual trapped bacteria is not constant in time but oscillates between two types of motion which broadly reproduce the bimodality observed in Figure 2f,g. Hence, we conclude that bimodality does not only arise from different cells swimming at different speeds, as Figure 2c–e may suggest, but that it also occurs for individual cells. We ascribe this observation to the presence of solid surfaces hindering flagellar bundling, which is amplified by the constriction imposed by the hydrodynamic trap. This results in cells spending prolonged lengths of time in a slow random walk state where they do not actively propel themselves (see SI 2 for the full analysis).

Overall, these results show that the measured motility signal is a combination of flagellar motility, micro‐scale motions due to metabolic activity, and background factors such as Brownian diffusion and flow‐induced motion. However, flagellar motility produces a much larger signal that dominates our signature. The low‐amplitude fluctuations produced by nonmotile bacteria are above the Brownian diffusion and flow‐dominated motion of dead cells and can be therefore considered fingerprints of bacterial viability.

### 2.2 Heteroresistance in an Engineered Polyclonal Population

Having verified that the assay is able to quantify and characterize the heterogeneity of bacterial motility, we now examine the effect of antibiotics. We start by creating *polyclonal* populations by mixing susceptible (S) and resistant (R) *E. coli* MG1655 in different ratios and following the motility distributions of the mixed population over time in the presence of 10 μg mL^−1^ kanamycin (a bactericidal antibiotic). Kanamycin‐resistant strains were prepared by transforming wild‐type *E. coli* MG1655 with a plasmid encoding a kanamycin‐resistant gene followed by selection on appropriate agar plates (see experimental details in SI 1.4 and SI 1.5).

**Figure 3:**
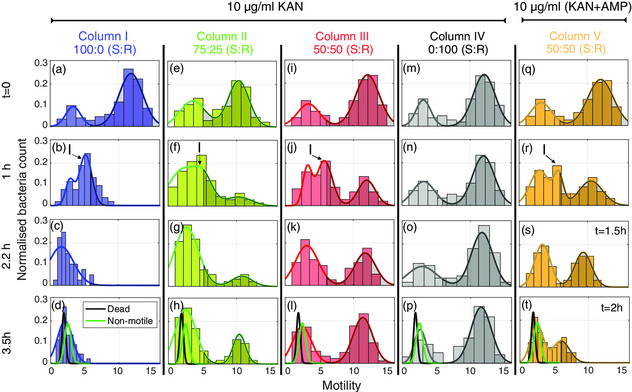
Motility histograms for different polyclonal heteroresistant *E. coli* populations over time. a–d) 100:0 susceptible:resistant (S:R) *E. coli* population. e–h) 75:25 S:R E. coli‐mixed population. i–l) 50:50 S:R E. coli‐mixed population. e–h) 0:100 S:R E. coli population. In all cases, 10 μg mL^−1^ kanamycin was used. q–t) 50:50 S:R E. coli‐mixed population exposed to 10 μg mL^−1^ kanamycin and 10 μg mL^−1^ of ampicillin (both bactericidal antibiotics). Continuous lines represent unimodal, bimodal, or trimodal Gaussian fits depending on the conditions. The green and black Gaussian fits in panels (d), (h), (l), (p), and (t) represent distributions of dead and nonmotile bacteria included as a reference. The gray shaded regions beneath the Gaussian fits in all histograms represent a 50% motility threshold defining two subpopulations of different motility (i.e., low and high motility, respectively). The ‘I’ in panels (b), (f), (j), and (r) indicates transient intermediate‐motility peaks.

Experimental results are shown in **Figure** **3**, where each panel represents a histogram of measured motility at a given time point (different rows), for four different bacterial populations (different columns). The histograms at *t* = 0 (first row) report cumulative counts of no‐antibiotic controls where bacteria were observed in the absence of kanamycin. Kanamycin is then administered shortly after *t* = 0. In the case of a fully susceptible population (Column I of Figure 3), we observe a significant change in motility over time. The motility starts as a bimodal distribution at *t* = 0, then becomes weakly bimodal after 1 h (second row), and finally turns into unimodal distribution after 2.2 h (third and fourth rows of Column I). In fact, the final distribution at *t* = 3.5 h overlaps with the nonmotile and dead populations, suggesting that flagellar motility is almost completely suppressed by kanamycin at this point. In contrast, the purely kanamycin‐resistant population (Column IV) does not show any significant change in motility over time. These results not only confirm the kanamycin resistance of the transformed wild‐type strain, but they also confirm that the time‐dependent loss of motility is an effective measure of susceptibility to this bactericidal antibiotic.

**Figure 4:**
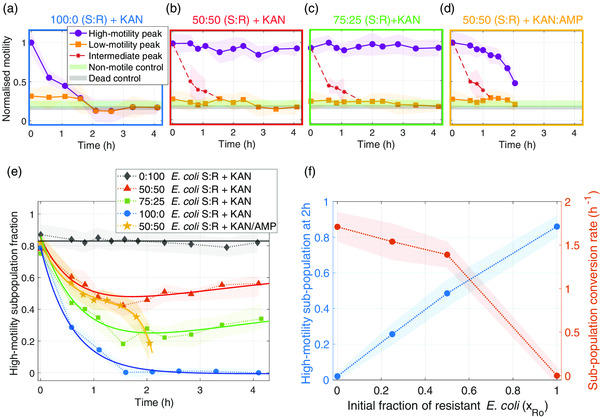
Motility of each subpopulation for different polyclonal heteroresistant *E. coli* populations. a–d) Time evolution of the motility of each subpopulation for different initial compositions of the population. The green and black shaded areas represent the motility of dead and nonmotile bacteria. e) High‐motility subpopulation fraction *f*_1_ over time for all of the mixed population experiments. Continuous lines represent best fits to an exponential logistic model derived in SI 4. f) (left‐hand side (LHS) axis) Value of the high‐motility subpopulation fraction after 2 h of exposure to kanamycin as a function of the initial fraction of resistant bacteria in the sample. (right‐hand side (RHS) axis) Exponential decay time constant (1/t* in Equation S2, Supporting Information) obtained from fitting the curves in panel. e) Shaded areas in all panels represent standard deviation based on two replicates (for the 50:50 S:R + KAN/AMP case) or three replicates (for all other cases).

Heteroresistant populations were obtained by mixing S and R *E. coli* in different ratios, specifically 75:25 (Column II, Figure 3) and 50:50 (Column III, Figure 3). In both cases, after about 1 h exposure to kanamycin (second row), we detect a bacterial subpopulation that exhibits intermediate motility (most notably in panel (j), labelled with “I”). This subpopulation represents the fraction of susceptible bacteria in the mix, as it follows the same trend as the fully susceptible population (see also **Figure** **4**a–c below). Similarly, the high‐motility peaks represent the fraction of resistant bacteria in the mix and this fraction continues to grow in the presence of the antibiotic, most notable in the bottom panels of Columns II and III.

To further highlight the versatility of our approach, we exposed the 50:50 population of Column III to a mix of antibiotics, here kanamycin and ampicillin, as shown in Column V of Figure 3. We note that the introduction of ampicillin inhibited the motility of the kanamycin‐resistant subpopulation, as evidenced by the high‐motility peak decreasing both in amplitude and in average value over time, until most cells lysed due to the ampicillin‐induced weakening of the bacterial cell wall. We have also independently tested ampicillin on fully susceptible and resistant populations to confirm that ampicillin also inhibits motility (see results in SI 3). Using a cocktail of antibiotics is common clinical practice, and by observing a markedly different response compared with a single antibiotic, we confirm the viability of our approach for both mixed bacterial populations and for mixtures of different antibiotics (for additional detail, see SI 1 and SI 3).

### 2.3 Quantification of Heteroresistance in Polyclonal Populations

The data presented in Figure 3 are very information rich, but the motility histograms are mainly a qualitative comparison of different heterogeneous bacterial populations. We now provide a more quantitative analysis of these data by plotting the motility of each subpopulation as a function of time (Figure 4a–d). The first observation is that the exponential decay of the high‐motility peak for the 100% susceptible population (Figure 4a) is very closely mirrored by the behavior of the intermediate populations. This is illustrated by the red dashed curves in Figure 4b–d, which show the time evolution of the intermediate (“I”) peaks from Columns II, III, and V of Figure 3. This confirms our expectation that the susceptible subpopulations behave exactly as the pure populations. This is unsurprising, but it is important to note that our method can clearly identify these subpopulations.

In contrast, bacteria in the slow random walk state (i.e., low‐motility peak) appear weakly affected by the action of the antibiotic, as suggested by their delayed loss of motility evidenced by the orange curves in Figure 4a–c. It has previously been demonstrated that antibiotics alter the nanomechanical vibrations of susceptible bacteria.^[^ ^23^, ^24^ ^]^ However, the magnitude of these vibrations is an order of magnitude smaller than the motion that we can reproducibly detect in our device. Therefore, it is likely that the low‐motility bacteria are affected by the antibiotic, but their change in motion is not observable reliably in our assay.

Next, we consider the fraction of high‐motility subpopulation alone by calculating the areas under the Gaussian fits for motility values higher that 50% of the motility measured in the absence of antibiotic (i.e., gray shaded regions in histograms of Figure 3). We then fit the time‐dependent change in the areas as the sum of exponential and logistic terms (continuous lines in Figure 4e). The exponential term models the decrease in motility due to the bactericidal effects of kanamycin on susceptible cells, while the sigmoidal logistic term accounts for the multiplying resistant bacteria (a full derivation of the model is presented in SI 4).

**Figure 5:**
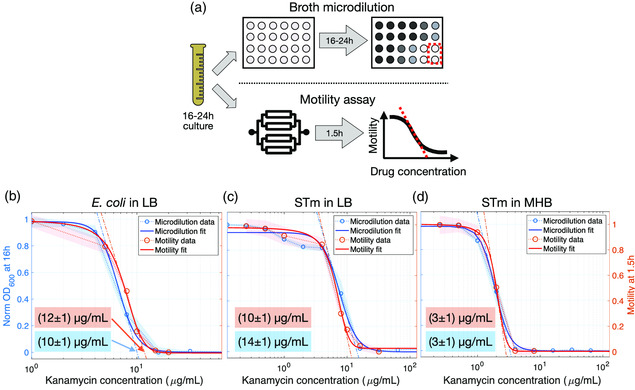
Comparison between the motility assay and the broth microdilution methods. a) Schematic illustration of microdilution plate‐based and microfluidic chip motility assays. Starting from an overnight bacterial suspension, the microdilution plate‐based method produces a value of the MIC by measuring the OD of bacterial suspension after 16 h of incubation. In the microfluidic chip‐based assay, the motility is measured after 1.5 h of exposure to kanamycin within the device. b,c,d) Normalized OD (blue circles) measured after 16 h of incubation and normalized motility (red circles) after 1.5 h of exposure to increasing kanamycin concentrations of (b) *E. coli* in LB, c) *S. Typhimurium* (STm) in LB, and d) STm in MHB. Shaded areas denote standard deviation based on six replicates (OD, blue areas) and three replicates (motility, red areas). The continuous lines are best‐fit curves to the sigmoidal Gompertz function for both OD and motility. The MIC values are obtained as the *x*‐axis intercept of the tangent at the inflection point of the fitted curves (dashed–dotted lines) and reported in the corresponding color‐coded inset boxes.

Two important parameters can be extracted from the resulting curves: the subpopulation conversion rate and the magnitude of the fitting function at *t* = 2 h (Figure 4f). The conversion rate captures the time‐dependent loss of motility in terms of a time constant for exponential decay (b), while the magnitude of the fitting function informs about the composition of the population after 2 h of antibiotic exposure. We observe a linear correlation between the high‐motility subpopulation and the initial fraction of resistant *E. coli* (blue curve in Figure 4f), which supports the key finding that heteromotility is an excellent reporter for heteroresistance. In this case, heteroresistance was engineered by mixing the polyclonal subpopulations, but the linearity of the curve indicates that the method could be readily calibrated for unknown populations. Furthermore, in the context of **Figure** **5**, we show that our motility method can also be used to identify heteroresistance in an unknown population. The exponential decay time constant (orange curve in Figure 4f) is also correlated with the initial fraction of resistant bacteria, thereby providing another fingerprint of the composition of the population.

In addition, the rate of motility loss can provide information about the mode of action of the antibiotic, as shown by a comparison between kanamycin, trimethoprim (a bacteriostatic antibiotic), and ampicillin (see SI 3). The comparison between these three antibiotics in SI 3, as well as our previous work,^[^ ^31^ ^]^ also demonstrates that motility is an independent metric of susceptibility, which is robust against changes in bacterial morphology, such as trimethoprim‐induced filamentation or the formation of V‐shaped bacteria following exposure to ampicillin.

### 2.4 Determination of the MIC

In the following experiments, we explored a different method to rapidly characterize an “unknown” bacterial sample and therefore show the diagnostic potential of our device. We investigated the dependence of motility on antibiotic concentration at a fixed time point to probe for heteroresistance. Fixing the measurement time point while varying the drug concentration provides a better insight into the level of resistance in the subpopulations and, as an added additional benefit, enables the simultaneous determination of the MIC. We first consider the determination of the MIC.

The motility of purely susceptible populations of *E. coli* MG1655 or *S. Typhimurium* at a fixed time point (1.5 h) was measured for twofold serial dilutions of kanamycin and in two different growth media (lysogeny broth [LB] and Müller‐Hinton broth [MHB]). Results from the motility assay were compared with a traditional growth assay (i.e., the broth microdilution method), as illustrated schematically in Figure 5a. For both procedures, we initially selected a bacterial isolate from an agar plate, suspended it in growth medium, and allowed it to incubate overnight at 37 °C. It is worth highlighting that most microfluidic platforms require the first culturing step followed by dilution to a suitable optical density (OD),^[^ ^22^, ^23^, ^24^, ^25^, ^26^, ^39^, ^40^ ^]^ even though this aspect may not be explicitly emphasized. This is often the case unless an enrichment step is integrated in the workflow or the microfluidic device itself.^[^ ^41^, ^42^ ^]^ After this first step, a typical large‐scale growth assay requires further overnight incubation at 37 °C in the presence of the antibiotic to make changes in the OD induced by the drug detectable. In contrast, our motility assay eliminates the need for the second, long incubation step. Instead, after dilution into a fresh medium, the culture is simply spiked with the antibiotic and injected directly into the microfluidic device.

The motility is then measured after only 1.5 h of exposure to kanamycin. The normalized OD for the growth assay after 16 h and the normalized motility values after 1.5 h are provided in Figure 5b–d (refer to SI 1 for additional experimental details). The similarity of the curves for both assays is striking. It indicates that kanamycin inhibits both growth and motility according to a similar dose‐dependent pattern. Both curves were fit to a Gompertz function^[^ ^43^ ^]^ where the tangent is normally used to determine the MIC.^[^ ^23^, ^43^ ^]^ Note that the resulting MIC values for both assays are in very close agreement, as indicated in the insets of Figure 5b–d (see SI 1.3 for further details). This excellent agreement confirms that single‐cell motility is a viable proxy for colony‐level growth as used in traditional, low‐throughput antibiotic susceptibility tests.

### 2.5 Heteroresistance in a Monoclonal Population

When examining Figure 5c for *S. Typhimurium* in LB in more detail, it is apparent that the curves for both growth and motility deviate from the predicted sigmoidal dependence at a concentration of around 1 μg mL^−1^, which is much lower than the calculated MIC (14 μg mL^−1^). This shows that some fraction of the *S. Typhimurium* population is already affected at this lower antibiotic concentration, while the remainder of the population is not. Such behavior is atypical, considering that the population is monoclonal (i.e., genetically identical) for which one would expect a much sharper decrease at an antibiotic concentration two‐ to fourfold lower than the MIC^[^ ^5^, ^6^ ^]^ (e.g., as observed in Figure 5d for the same strain of *S. Typhimurium* cultured in MHB).

Such a wide range of growth inhibitions spanning an order of magnitude of concentration, in conjunction with the biphasic pattern, suggests that *S. Typhimurium* grown in LB is heteroresistant to kanamycin. Here, we adopt a recent definition of the term heteroresistance^[^ ^5^, ^6^, ^7^ ^]^ in which a bacterial population is assumed to be heteroresistant if the MIC is more than eight times higher than the lowest noninhibitory concentration. The eightfold threshold criterion is necessary to ensure that one is not simply describing subpopulations with only marginal changes in the MIC, which are commonly found in most isolates.^[^ ^6^ ^]^ In other words, partial growth inhibition occurring over a sufficiently large range of concentrations can be considered as a signature of the presence of distinct subpopulations with significantly different levels of resistance. The biphasic trend we observe (Figure 5c) adds further weight to this argument. To confirm the observation of heteroresistance, we also conducted a traditional PAP test on agar plates (as described in SI 1.6). Overall, both the PAP and the growth assay confirm the capability of our motility assay to detect heteroresistance even for a monoclonal population, further supporting the use of motility as a viable proxy for bacterial growth and antibiotic susceptibility.

A comparison of the data in Figure 5c,d also reveals two intriguing observations. Firstly, *S. Typhimurium* cultured in LB displays heteroresistance to kanamycin. The *S. Typhimurium* cells originated from a single colony grown on an agar plate; therefore, the population is isogenic. Nevertheless, it is well known that a genetically identical population of cells does not necessarily have a homogeneous phenotype, because the intrinsic stochasticity of gene expression can result in subpopulations with a different phenotype that may in turn confer adaptive advantages to a population under stress. For example, Sanchez‐Romero *et al*.^[^ ^44^ ^]^ observed that an isogenic population of *S. Typhimurium* strain SL1344 showed heterogeneous population‐wide expression of the *ompC* gene, which encodes an abundant outer membrane porin. As aminoglycoside antibiotics such as kanamycin gain entry to Gram‐negative bacteria through porin channels in their outer membrane,^[^ ^45^, ^46^ ^]^ the reduced expression of *ompC* in some cells contributes to an increased resistance to the antibiotic.^[^ ^47^ ^]^

Second, the *S*. *Typhimurium* heteroresistance is only observed when cells are grown in LB. This may be due to the difference in osmolarity between the two media. It is known that the level of expression for porin genes and the relative amounts of porins with different channel sizes in the outer membrane strongly depend on pH, osmolarity, nutrient availability, and temperature of the medium.^[^ ^48^ ^]^ Considering that pH and temperature were constant across all experiments, and that both media types can normally support rich bacterial growth, it suggests that the observed heteroresistance in LB may be due to differences in osmolarity. High osmolarity has been linked to an increased production of the narrow OmpC porin compared with the larger OmpF porin in *E. coli* and *S. Typhimurium*.^[^ ^49^ ^]^ Considering that LB has a higher monovalent salinity than MHB, a likely explanation is that *S. Typhimurium* cells in LB produce a comparatively higher fraction of the narrow OmpC porin, through which kanamycin uptake is hindered. In conjunction with the aforementioned different expression levels for the *ompC* and *ompF* genes occurring in individual cells, this may favor the formation of a resistant subpopulation featuring reduced kanamycin uptake.

Overall, these results show that the reduction in motility of *E. coli* and *S.* Typhimurium exposed to kanamycin is directly correlated with the inhibition of bacterial growth and therefore, to the gold‐standard method for assessing antibiotic susceptibility. Crucially, the inhibition of motility manifests quickly and can be clearly detected after only 1.5 h of observation at the single‐cell level, as opposed to >16 h, as required by large‐scale bacterial growth assays. In addition, given the remarkable similarity between the measured trends, we suggest that bacterial motility can be used instead of growth to rapidly quantify the MIC and detect monoclonal or polyclonal heteroresistance.

## 3 Discussion and Conclusion

Heterogeneity dominates the observed motility distributions of bacilli such as *E. coli* and *S. Typhimurium*. This heterogeneity is observed both at the single‐cell level, meaning that trapped bacteria can occupy two distinct motility states, and at the population level, as highlighted by a small fraction of cells that consistently swim slower than the rest of the population. These observations align with the broader concept of phenotypical heterogeneity that bacterial populations may use as a strategy for maximizing population‐wide fitness.

The single‐cell resolution provided by hydrodynamic trapping enables characterization of heteroresistance in both monoclonal and polyclonal bacterial communities. In the case of *E. coli*, we used polyclonal populations consisting of genetically distinct *E. coli* obtained by mixing wild‐type susceptible cells and transformed resistant cells. Such a scenario emulates a situation where heterogeneity may arise in mixed infections (i.e., infections caused by strains of different resistances), as has been observed for example with *Mycobacterium tuberculosis* ^[^ ^50^ ^]^ or *Helicobacter pylori*.^[^ ^51^ ^]^

In the case of such mixed populations, we first noted a rapid exponential loss of motility caused by kanamycin acting on the susceptible portion of the *E. coli* population, while the remaining motile bacteria are a fingerprint of a resistant subpopulation that continued to swim and divide in the presence of the antibiotic. Measuring this fingerprint only requires a 2 h observation of swimming cells in the presence of kanamycin. The uptake of aminoglycoside antibiotics like kanamycin is known to occur in three phases.^[^ ^46^ ^]^ It is not until the last phase that RNA misreading and consequent cell death is initiated and the accompanying lag time has been estimated to be in the range of 30–60 min.^[^ ^52^, ^53^ ^]^ This lag time is consistent with the timescale over which the high‐motility subpopulation loses its swimming ability (Figure 4e). We also verified that the addition of ampicillin inhibited the proliferation of the kanamycin‐resistant subpopulation, thereby demonstrating that our motility assay can measure the action of antibiotic cocktails, as well as antibiotics with different mechanisms of action (SI 3). The latter attribute was demonstrated using our hydrodynamic trapping approach to detect susceptibility to bacteriostatic antibiotics like trimethoprim (see SI 3.1), which induces a more gradual loss of motility due to filamentation rather than cell death.

In the case of *S. Typhimurium*, we detected monoclonal heteroresistance (i.e., heteroresistance in a population of genetically identical clones). The presence of heteroresistance was detected as a biphasic inhibition of motility observed over a large range of kanamycin concentrations below the MIC. Remarkably, the inhibition of large‐scale growth induced by the same range of concentrations follows an identical trend, thus demonstrating the suitability of single‐cell motility for detecting monoclonal heteroresistance after only 1.5 h of exposure to kanamycin. The presence of heteroresistance was also confirmed by a standard PAP test conducted using agar plates. The observed monoclonal heteroresistance could be explained by the heterogeneous expression of porin genes as also suggested in a previous study.^[^ ^44^ ^]^ This heterogeneity, possibly in conjunction with other epigenetic factors (e.g., higher osmolarity of LB favors production of the narrower OmpC channel), could contribute to the establishment of a subpopulation which is more resilient to kanamycin because of reduced drug uptake. It is unlikely that the monoclonal heteroresistant population had acquired low‐level kanamycin resistance through mutations, as these are typically observed at much lower frequencies.^[^ ^44^ ^]^

Overall, the key insight that this study offers is that bacterial motility can directly and rapidly report on bacterial heteroresistance and antibiotic susceptibility. As a consequence, motility can be used as a valuable high‐throughput parameter to characterize certain types of heteroresistance at the single‐cell level. Single‐cell motility and growth are also affected with remarkably similar dose‐dependent patterns. This similarity is a powerful finding that allows for motility to be used as a rapid phenotypic measure of the MIC of bacteriostatic and bactericidal antibiotics. Ultimately, these capabilities arise from the fact that changes in motility due to the antibiotic treatment happen on a much shorter timescale (<2 h) compared with bulk bacterial growth (>16 h). By conducting high‐throughput single‐cell measurements, changes to the motility phenotype are detected as soon as they happen. In contrast, in existing bulk assays, the sample heterogeneity is masked by the spatial or temporal averaging inherent to the data acquisition procedures, which also extend the time needed for phenotypical changes to be detected. This means that our method is limited by the biological timescale of the antibiotic action on motility, rather than by the need to wait for detectable cell growth.

We envisage that a microfluidic, motility‐sensing device could rapidly quantify the MIC, while simultaneously detecting monoclonal or polyclonal heteroresistance in a bacterial sample. Our assay is mainly applicable to motile bacterial strains, given that flagellar motor‐driven motility is used as the indicator of susceptibility. However, we note that flagellated motility is a virulence factor in many pathogenic bacteria and has a significant role in colonization, such as with UTIs.^[^ ^27^, ^28^, ^29^, ^30^ ^]^ The geometry of the hydrodynamic traps can be selected for a given cell shape,^[^ ^31^, ^34^ ^]^ so that our platform could also be tuned to different bacterial morphologies. We have shown some examples of elongated and V‐shaped *E. coli* following treatment with trimethoprim and ampicillin, respectively, thus demonstrating the applicability of our platform to different cellular morphologies (see SI 3). Multiple devices could be used in parallel to further increase sample throughput, while providing a massive reduction in material handling requirements and processing time. These design features could be integrated into future drug screening or point‐of‐care diagnostics, where changes in single‐cell motility could be monitored alone as or in conjunction with other parameters like cell morphology or metabolism.

## 4 Experimental Section

4.1

4.1.1

##### Traps and Microfluidic Chip Fabrication

The hydrodynamic traps were fabricated in the electron‐beam resist SU8 on microscope slides following the protocol we previously developed.^[^ ^31^ ^]^ The microfluidic channel was fabricated in polydimethylsiloxane (PDMS) with a SU8 mold on silicon by crosslinking a mixture of silicon elastomer and curing agent (Dow Corning) in a 7:1 ratio. The mixture was baked for 12–16 h at 60 °C before being peeled off the mold and bonded to the previously fabricated trapping arrays by treating both surfaces with O_2_ plasma for 2 min at a flow of 5 standard cubic centimeters per minute (sscm).

##### Culturing and Transformation of Bacterial Strains

Aliquots from frozen glycerol stocks of *E. coli* MG1655, *E. coli* BW25113, and *Salmonella enterica enterica* serovar Typhimurium strain LT2 were plated onto LB, Miller formulation, agar plates and incubated overnight at 37 °C. Single colonies were picked from the plates and suspended in LB or MHB depending on the experiment and incubated at 37 °C overnight in static conditions, before being diluted into fresh LB or MHB to a concentration of ≈10^7^ CFU mL^−1^. Antibiotic resistant *E. coli* were prepared by transforming strains via electroporation with plasmid DNA encoding the appropriate antibiotic resistance gene cassette, and transformants were selected on agar plates supplemented with the appropriate antibiotic (i.e., 100 μg mL^−1^ ampicillin, 30 μg mL^−1^ kanamycin). Dead bacteria were obtained by heat inactivation at 65 °C in a block heater. More details about bacterial transformations, selection, mixing, growth curve measurements, and antibiotic serial dilutions for the determination of the MIC can be found in the Supplementary Information.

##### Hydrodynamic Trapping Experiments

Prior to bacteria injection, the assembled microfluidic chips were flushed with a 1% (w/v) solution of bovine serum albumin (BSA) in phosphate‐buffered saline (PBS) to prevent bacterial attachment. The BSA solution was injected at 30 μL min^−1^ and then pumped at 1 μL min^−1^ for at least 30 min before each experiment. Bacterial solutions (≈300 μL) were then injected at 15 μL min^−1^ before decreasing the flow rate to 10–15 nL min^−1^. A microfluidic syringe pump (LEGATO 180, KD Scientific) was used throughout all the experiments. Videos of the trapping experiment were recorded with a DSLR camera (Nikon D3300) using a Leica phase contrast microscope (Leica DM IRB) equipped with a 60× magnification objective (Leica, NA 0.7). Videos were recorded at 50 fps and were typically 3–5 min in length.

##### Data Analysis

Videos were processed using a custom, semiautomated MATLAB script that retrieved the average pixel intensity as a function of time from ROIs centered within each hydrodynamic trap for a wide‐field image (240 μm × 135 μm) of the entire trapping array. Intensity traces from these ROIs were processed with the same MATLAB script to calculate the standard deviation in a given observation window for a trapped bacterium (*σ*_trapped_) and the corresponding empty trap (*σ*_empty_). Subsequent data analysis, Gaussian and Gompertz fits, and error calculations were all performed with standard MATLAB functions. All experiments were repeated at least in duplicate, unless otherwise stated, and the standard error of the average (*σ*_trapped_/*σ*_empty_) ratio was reported for each experimental condition. More details about some of these procedures can be found in the Supplementary Information.

## Conflict of Interest

The authors declare no conflict of interest.

## Supporting information

Supplementary Material

## Data Availability

The data that support the findings of this study are available from the corresponding author upon reasonable request.

## References

[1] M. E. Lidstrom , M. C. Konopka , Nat. Chem. Biol. 2010, 6, 705. 20852608 10.1038/nchembio.436

[2] K. M. Davis , R. R. Isberg , BioEssays 2016, 38, 782. 27273675 10.1002/bies.201500121

[3] N. Eling , M. D. Morgan , J. C. Marioni , Nat. Rev. Genet. 2019, 20, 536. 31114032 10.1038/s41576-019-0130-6 PMC7611518

[4] X. Wang , Y. Kang , C. Luo , T. Zhao , L. Liu , X. Jiang , R. Fu , S. An , J. Chen , N. Jiang , L. Ren , Q. Wang , J. Kenneth Bailli , Z. Gao , J. Yu , MBio 2014, 5, 1. 10.1128/mBio.00942-13 PMC3950525 24520060

[5] O. M. El-Halfawy , M. A. Valvano , Clin. Microbiol. Rev. 2015, 28, 191. 25567227 10.1128/CMR.00058-14 PMC4284305

[6] D. I. Andersson , H. Nicoloff , K. Hjort , Nat. Rev. Microbiol. 2019, 17, 479. 31235888 10.1038/s41579-019-0218-1

[7] V. I. Band , E. K. Crispell , B. A. Napier , C. M. Herrera , G. K. Tharp , K. Vavikolanu , J. Pohl , T. D. Read , S. E. Bosinger , M. S. Trent , E. M. Burd , D. S. Weiss , Nat. Microbiol. 2016, 1, 16053. 27572838 10.1038/nmicrobiol.2016.53 PMC5154748

[8] H. Nicoloff , K. Hjort , B. R. Levin , D. I. Andersson , Nat. Microbiol. 2019, 4, 504. 30742072 10.1038/s41564-018-0342-0

[9] G. Pitruzzello , D. Conteduca , T. F. Krauss , Nanophotonics 2020, 9, 4447.

[10] V. I. Band , S. W. Satola , E. M. Burd , M. M. Farley , J. T. Jacob , D. S. Weiss , MBio 2018, 9, e02448. 29511071 10.1128/mBio.02448-17 PMC5844991

[11] Y.‐Y. Liu , Y. Wang , T. R. Walsh , L.‐X. Yi , R. Zhang , J. Spencer , Y. Doi , G. Tian , B. Dong , X. Huang , L.‐F. Yu , D. Gu , H. Ren , X. Chen , L. Lv , D. He , H. Zhou , Z. Liang , J.‐H. Liu , J. Shen , Lancet Infect. Dis. 2016, 16, 161. 26603172 10.1016/S1473-3099(15)00424-7

[12] S. W. Satola , M. M. Farley , K. F. Anderson , J. B. Patel , J. Clin. Microbiol. 2011, 49, 177. 21048008 10.1128/JCM.01128-10 PMC3020420

[13] H. Yin , D. Marshall , Curr. Opin. Biotechnol. 2012, 23, 110. 22133547 10.1016/j.copbio.2011.11.002

[14] S. M. Prakadan , A. K. Shalek , D. A. Weitz , Nat. Rev. Genet. 2017, 18, 345. 28392571 10.1038/nrg.2017.15 PMC5495114

[15] P. Wang , L. Robert , J. Pelletier , W. L. Dang , F. Taddei , A. Wright , S. Jun , Curr. Biol. 2010, 20, 1099. 20537537 10.1016/j.cub.2010.04.045 PMC2902570

[16] S. Taheri-Araghi , L. Robert , J. Pelletier , W. L. Dang , F. Taddei , A. Wright , S. Jun , Curr. Biol. 2015, 25, 385.25544609

[17] T. M. Norman , N. D. Lord , J. Paulsson , R. Losick , Nature 2013, 503, 481. 24256735 10.1038/nature12804 PMC4019345

[18] N. D. Lord , T. M. Norman , R. Yuan , S. Bakshi , R. Losick , J. Paulsson , Science (80-.). 2019, 366, 116. 10.1126/science.aaw4506 PMC7526939 31604312

[19] N. Q. Balaban , Science (80-.). 2004, 305, 1622. 10.1126/science.1099390 15308767

[20] F. Lyu , M. Pan , S. Patil , J.‐H. Wang , A.C. Matin , J. R. Andrews , S. K.Y. Tang , Sensors Actuators, B Chem. 2018, 270, 396.

[21] O. Scheler , K. Makuch , P. R. Debski , M. Horka , A. Ruszczak , N. Pacocha , K. Sozański , O.‐P. Smolander , W. Postek , P. Garstecki , Sci. Rep. 2020, 10, 1. 32094499 10.1038/s41598-020-60381-z PMC7039976

[22] A. Rohani , J. H. Moore , Y.‐H. Su , V. Stagnaro , C. Warren , N. S. Swami , Sensors Actuators, B Chem. 2018, 276, 472. 10.1016/j.snb.2018.08.137 PMC6201234 30369719

[23] G. Longo , L. Alonso-Sarduy , L. M. Rio , A. Bizzini , A. Trampuz , J. Notz , G. Dietler , S. Kasas , Nat. Nanotechnol. 2013, 8, 522. 23812189 10.1038/nnano.2013.120

[24] H. Etayash , M. F. Khan , K. Kaur , T. Thundat , Nat. Commun. 2016, 7, 12947. 27698375 10.1038/ncomms12947 PMC5059454

[25] V. Kara , C. Duan , K. Gupta , S. Kurosawa , D. J. Stearns-Kurosawa , K. L. Ekinci , Lab Chip 2018, 18, 743. 29387860 10.1039/c7lc01019b PMC5829026

[26] K. Syal , R. Iriya , Y. Yang , H. Yu , S. Wang , S. E. Haydel , H.‐Y. Chen , N. Tao , ACS Nano 2016, 10, 845. 26637243 10.1021/acsnano.5b05944

[27] C. Josenhans , S. Suerbaum , Int. J. Med. Microbiol. 2002, 291, 605. 12008914 10.1078/1438-4221-00173

[28] M. C. Lane , V. Lockatell , G. Monterosso , D. Lamphier , J. Weinert , J. R. Hebel , D. E. Johnson , H. L. T. Mobley , Infect. Immun. 2005, 73, 7644. 16239569 10.1128/IAI.73.11.7644-7656.2005 PMC1273871

[29] B. Chaban , H. V. Hughes , M. Beeby , Semin. Cell Dev. Biol. 2015, 46, 91. 26541483 10.1016/j.semcdb.2015.10.032

[30] Q. Duan , M. Zhou , L. Zhu , G. Zhu , J. Basic Microbiol. 2013, 53, 1. 22359233 10.1002/jobm.201100335

[31] G. Pitruzzello , S. Thorpe , S. Johnson , A. Evans , H. Gadêlha , T. F. Krauss , Lab Chip 2019, 9, 1417. 10.1039/c8lc01397g 30869093

[32] D. Di Carlo , L. Y. Wu , L. P. Lee , Lab Chip 2006, 6, 1445. 17066168 10.1039/b605937f

[33] C. Probst , A. Grünberger , W. Wiechert , D. Kohlheyer , Micromachines 2013, 4, 357.

[34] M.‐C. Kim , B. C. Isenberg , J. Sutin , A. Meller , J. Y. Wong , C. M. Klapperich , Lab Chip 2011, 11, 1089. 21293825 10.1039/c0lc00362j

[35] T. K. Wood , A. F. González Barrios , M. Herzberg , J. Lee , Appl. Microbiol. Biotechnol. 2006, 72, 361. 16397770 10.1007/s00253-005-0263-8

[36] C. V. Gabel , H. C. Berg , Proc. Natl. Acad. Sci. U. S. A. 2003, 100, 8748. 12857945 10.1073/pnas.1533395100 PMC166384

[37] C. R. Bermingham , I. Murillo , A. D. J. Payot , K. C. Balram , M. B. Kloucek , S. Hanna , N. M. Redmond , H. Baxter , R. Oulton , M. B. Avison , M. Antognozzi , bioRxiv 2018, 460139, 10.1101/460139.

[38] K. Syal , S. Shen , Y. Yang , S. Wang , S. E. Haydel , N. Tao , ACS Sensors 2017, 2, 1231. 28741927 10.1021/acssensors.7b00392

[39] Ö. Baltekin , A. Boucharin , E. Tano , D. I. Andersson , J. Elf Proc. Natl. Acad. Sci. 2017, Vol. 114, p. 9170. 28790187 10.1073/pnas.1708558114 PMC5576829

[40] Y. Yang , K. Gupta , K. L. Ekinci , Proc. Natl. Acad. Sci. 2020, 117, 10639. 32350139 10.1073/pnas.1922172117 PMC7245095

[41] I. Michael , D. Kim , O. Gulenko , S. Kumar , S. Kumar , J. Clara , D. Y. Ki , J. Park , H. Y. Jeong , T. S. Kim , S. Kwon , Y.‐K. Cho , Nat. Biomed. Eng. 2020, 4, 591. 32424198 10.1038/s41551-020-0557-2

[42] T. N. T. Dao , J. Yoon , C. E. Jin , B. Koo , K. Han , Y. Shin , T. Y. Lee , Sensors Actuators, B Chem. 2018, 262, 588.

[43] R. J. W. Lambert , J. Pearson , J. Appl. Microbiol. 2000, 88, 784. 10792538 10.1046/j.1365-2672.2000.01017.x

[44] M. A. Sánchez-Romero , J. Casadesús Proc. Natl. Acad. Sci. U. S. A. 2014, 111, 355. 24351930 10.1073/pnas.1316084111 PMC3890857

[45] R. E. W. Hancock , J. Bacteriol. 1987, 169, 929. 2434461 10.1128/jb.169.3.929-933.1987 PMC211881

[46] H. W. Taber , J. P. Mueller , P. F. Miller , A. S. Arrow , Microbiol. Rev. 1987, 51, 439. 3325794 10.1128/mr.51.4.439-457.1987 PMC373126

[47] O. Agafitei , E. J. Kim , T. Maguire , J. Sheridan , J. Exp. Microbiol. Immunol. 2010, 14, 34.

[48] H. Nikaido , Microbiol. Mol. Biol. Rev. 2003, 67, 593. 14665678 10.1128/MMBR.67.4.593-656.2003 PMC309051

[49] H. Nikaido , M. Vaara , Microbiol. Rev. 1985, 49, 1. 2580220 10.1128/mr.49.1.1-32.1985 PMC373015

[50] C. Zheng , S. Li , Z. Luo , R. Pi , H. Sun , Q. He , K. Tang , M. Luo , Y. Li , D. Couvin , N. Rastogi , Q. Sun , B. A. Forbes , J. Clin. Microbiol. 2015, 53, 2138. 25903578 10.1128/JCM.03507-14 PMC4473183

[51] M. T. Mascellino , B. Porowska , M. De Angelis , A. Oliva , Drug Des. Devel. Ther. 2017, 11, 2209. 10.2147/DDDT.S136240 PMC5546184 28814829

[52] L. E. Bryan , H. M. Van den Elzen , Antimicrob. Agents Chemother. 1976, 9, 928. 820248 10.1128/aac.9.6.928 PMC429653

[53] C. Hurwitz , C. B. Braun , C. L. Rosano , Biochim. Biophys. Acta—Nucleic Acids Protein Synth. 1981, 652, 168. 10.1016/0005-2787(81)90220-3 6163463

