# Supplementary material for: Single‐Cell Motility Rapidly Quantifying Heteroresistance in Populations of Escherichia coli and Salmonella typhimurium
Source: Small Sci. 2022 Mar 24;2(5):2100123. doi: 10.1002/smsc.202100123 (PMC11936031; doi:10.1002/smsc.202100123)
Supplement: Supplementary file 1 — Supplementary Material [file SMSC-2-2100123-s001.pdf]

Supplementary Information for

Single-cell motility can rapidly quantify heteroresistance in populations of E. coli and S. Typhimurium

Giampaolo Pitruzzello[1], Christoph G. Baumann[2], Steven Johnson[3], Thomas F. Krauss[1]*

[1] Department of Physics, University of York, YO105DD, York, UK

[2] Department of Biology, University of York, YO105DD, York, UK

[3] Department of Electronic Engineering, University of York, YO105DD, UK

*E-mail: giampaolo.pitruzzello@york.ac.uk

This PDF file includes:

Supplementary text

Figures S1 to S6

SI References

Supplementary Information Text

SI 1. Bacterial cultures, broth microdilution AST and PAP test

SI 1.1. 96-well plate and media preparation

For the microdilution AST, columns 1 and 12 of a standard sterile 96-well plate (GreinerBio 655185) were used as a no-antibiotic control, while columns 2-11 were used for a decreasing two-fold serial dilution of antibiotics. 100 µL of Lysogeny Broth (LB) or Müller-Hinton Broth (MHB) were first added to columns 1 to 12 of all the required rows in the plate.

For the antibiotic serial dilution, 100 µL of the 1024 µg mL^{-1} antibiotic solution was first added to column 2 (diluting the drug to 512 µg mL^{-1}), mixed by pipetting up-and-down in the well, then a 100 µL volume transferred to the next well and the process repeated to give a final concentration of 1 µg mL^{-1} in column 11. 100 µL of *E. coli* or *S*. Typhimurium suspension in the appropriate medium (LB or MHB) at a concentration of 10^6 CFU mL^{-1} (see below for details) was added to each well. This gave a bacterial concentration of $5x10^5$ CFU mL^{-1} and a series dilution from 256 µg mL^{-1} (column 2) to 0.5 µg mL^{-1} (column 11). Columns 1-4 of a separate row on the same plates contained 200 µL of LB or MHB as a sterility control.

Stock solutions of the antibiotics (50 mg mL^{-1}) were prepared from kanamycin sulphate (Sigma Aldrich K4000) and ampicillin sodium salt (Sigma Aldrich A0166). The antibiotic stock solutions were filter-sterilised through a 0.22 µm syringe filter before being diluted in sterile LB or MHB to 1024 µg mL^{-1}. A fresh solution was prepared before every experiment. The Miller formulation of LB was used throughout all the experiments (10 g L^{-1} tryptone, 10 g L^{-1} NaCl, 5 g L^{-1} yeast extract in deionised (DI) water, all from Sigma Aldrich). MHB was prepared by diluting 21 g of MHB powder (Sigma Aldrich 70192) in 1 L of DI water. All media were sterilised by autoclaving at 121 °C for 20 min and allowed to cool to room temperature prior to use.

SI 1.2. Bacteria preparation

Single colonies of *E. coli* MG1655 or *Salmonella enterica enterica* serovar Typhimurium strain LT2 (*S.* Typhimurium) were picked from agar plates and suspended in 3-5 mL of pure LB, MHB or LB supplied with the relevant antibiotic for plasmid selection (30 µg mL^{-1} kanamycin, 100 µg mL^{-1} ampicillin and 100 µg mL^{-1} trimethoprim), using a sterile 10 µL loop. Overnight incubations were carried out at 37 °C in static conditions using 50 mL screw-cap plastic tubes. Following overnight growth, the optical density (OD) at 600 nm was measured with a spectrophotometer (JENWAY 6300). The suspensions were diluted

in fresh LB to an OD of 0.1 to give a standardised concentration of ~10^8 CFU mL^{-1}. For the trapping experiments, a further 1:10 dilution was carried out, while for the broth microdilution experiments the OD = 0.1 suspensions were diluted 1:100 to obtain a concentration of ~10^6 CFU mL^{-1} which was then added to the wells as described above.

Growth at 37 °C in the 96-well plate was monitored by measuring the OD at 600 nm every 30 min using a plate reader (Biotek Synergy H1) over a period of 16 hours with orbital shaking at 200 RPM. The experiments were repeated in triplicate and in each replicate, all conditions were tested in duplicate or triplicate (i.e., each condition was realised in 2 or 3 of the 8 columns of each plate). The same procedure was repeated for the wild-type *E. coli* MG1655, the three transformed strains and *S.* Typhimurium in LB and MHB. The minimum inhibitory concentration (MIC) of the wild-type *E. coli* was determined to be (10 ± 1) μg/mL for kanamycin (see section S1.3 below), 1–2 μg mL^{-1} for trimethoprim and 4–8 μg mL^{-1} for ampicillin (data not shown). The same method confirmed the success of the transformation process which produced strains resistant to >256 μg mL^{-1} of each of the three drugs. The MIC of *S.* Typhimurium was found to be (14 ± 1) μg mL^{-1} in LB and (3 ± 1) μg mL^{-1} in MHB.

SI 1.3. MIC determination from OD data

As an example of the results produced from the microdilution assay, Figure S1(a) shows growth curves for the wild-type *E. coli* MG1655 in the presence of increasing concentrations of kanamycin in LB. Figure S1(b) shows the OD at 16 h as a function of the concentration of kanamycin and is the average over three biological replicates, each including two technical replicates. The values of OD are normalised to the range 0-1 in order to facilitate comparison with the motility curves in the main manuscript. The continuous line represents best-fit curve to the sigmoidal function:

$$OD(c) = OD_{low} + \frac{OD_{high}}{1+e^{\frac{c-c_h}{r}}} \quad \text{(eq. S1)}$$

where OD_{low} is the lower asymptote of the function (approximately equal to 0), OD_{high} is the distance between the upper and lower asymptote ($OD_{high} + OD_{low} \sim 1$), r is the slope of the decaying portion of the curve and c_h is the logarithm of the concentration at the inflection point.

SI 1.4. Bacterial transformation and plasmid selection

The following plasmids were used for the generation of antibiotic resistant *E. coli* MG1655: a pBAD vector containing ampR for ampicillin resistance, a pET-YSBLIC vector containing kanR for kanamycin resistance; and pT2ST containing ampR and dfrA for ampicillin and trimethoprim resistance, respectively.

For the transformation, *E. coli* MG1655 were grown overnight in LB at 37 °C. Cells were made electro-competent by washing an OD~0.6 suspension 3 times in ice-cold, sterile 10% (v/v) glycerol, before re-suspending in 100 µL of sterile 10% (v/v) glycerol. Electroporation was performed with a Bio-Rad Multipulse Electroporator using 50 µL of prepared cells and 40-150 ng of plasmid. Electroporated cells were added to 1 mL of LB warmed to 37 °C and then incubated at 37 °C for 2 h with shaking (220 RPM). 10 µL of culture was then plated onto either 100 µg mL^{-1} ampicillin or 30 µg mL^{-1} kanamycin LB agar plates and incubated at 37 °C for 24 h. Single colonies were picked from these plates to inoculate liquid cultures which were grown overnight with appropriate antibiotic selection as above and used to prepare glycerol stocks. The glycerol stocks were stored at -80 °C and used as the source of antibiotic resistant strains for all subsequent experiments. Single colonies were picked from freshly streaked plates (prepared using glycerol stock of appropriate bacterial strain) and transferred into 3-5 mL LB supplemented with the relevant antibiotic for plasmid selection, then incubated and used as described previously.

SI 1.5. Preparation of the mixed *E. coli* populations

The same incubation and dilution protocols were employed to grow bacterial samples for the hydrodynamic trapping experiments. Both the wild-type and transformed *E. coli* MG1655 strains were grown in the conditions specified above. Their OD at 600 nm was measured after the overnight incubation and both suspensions were diluted to a concentration of ~10^{8} CFU mL^{-1}. Different volumes of each suspension were re-suspended in fresh LB in order to obtain the 75:25 and 50:50 ratios of susceptible to resistant bacteria at a final (total) concentration of ~10^{7} CFU mL^{-1} before testing them in the hydrodynamic trapping assay.

SI 1.6. Population analysis profile test of *S.* Typhimurium

The population analysis profile (PAP) test was conducted to confirm heteroresistance of *S.* Typhimurium to kanamycin. LB agar plates were prepared by adding 15 g agar powder (Thermo Scientific 30391049) per litre of LB-Miller medium. After autoclaving at 121 °C for 20 min, the solutions were allowed to cool in a water bath at 43 °C. Different volumes of kanamycin stock solution were added to separate flasks to obtain a range of final concentrations and mixed well. The molten agar with kanamycin was then poured into petri dishes and allowed to cool until solidified.

S. Typhimurium were first grown overnight in liquid LB as described in SI 1.2. After overnight incubation, the suspension was adjusted to an OD = 0.1 and then diluted down by 1:10^6. 100 μL of 3 different dilution factors were dispensed on each kanamycin LB agar plate in duplicates and spread uniformly by using a sterile glass spreader. Plates were incubated for 24 h at 37 °C and colonies were counted manually (two or three times per plate) in order to quantify the fraction of surviving bacteria for each concentration of kanamycin. The results are shown in Figure S2, which illustrates that the inhibition of growth occurs over >8-fold range of kanamycin dilutions. In addition, note the bi-phasic behaviour of the curve, which reproduces the behaviour observed for the OD and motility in the main manuscript (Figure 7(c)) and suggests the existence of two sub-populations with different sensitivities to kanamycin.

SI 2. Single-cell vs population-wide heterogeneity

This section discusses the nature of the observed bimodality in the motility distributions of motile *E. coli* and *S.* Typhimurium (Figure 2 in the main manuscript). In order to understand its origin, it is necessary to explore the bacterial swimming phenotype. In a bulk solution, peritrichous bacteria such as *E. coli* and *S.* Typhimurium use flagellar bundles to propel themselves along straight running trajectories, until one (or more) flagellar motor switches its direction of rotation. When this happens, the flagella unbundle and the cell tumbles, resulting in a short phase of erratic movement at low speed until the motor(s) rotating in reverse switches direction again, followed by flagella re-bundling and initiation of a new run, typically along a different direction[1].

To verify whether cells confined to our hydrodynamic traps still employ this swimming strategy, we analysed the intensity traces produced by individual trapped bacteria. Typical examples are shown in Figure S3. Upon close inspection, it is evident that the standard deviation of the traces is not constant in time. This inhomogeneity is reflected in the behaviour of the moving standard deviation (calculated over a 5 s sliding window) shown in the second column of Figure S3 and quantified by the corresponding probability density functions (PDFs) in the third column. Notably, most cells show bimodal PDFs that broadly follow the population-wide distributions observed in Figure 2 in the main manuscript. This suggests that a single bacterium can occupy two distinct motility states that are maintained for extended periods of time.

This behaviour would not be expected if cells were swimming via the characteristic run-and-tumble pattern. We reason that bulk run-and-tumble would not produce the strong degree of bimodality observed here since the characteristic duration of tumbling events, during which cells do not actively propel themselves, is of the order of 140 ms[1,2]. In contrast, we observe that cells can spend up to several seconds

in the low-motility state, as clearly seen from the moving standard deviations in the second column of Figure S3.

Therefore, an alternative mechanism must be at play and interfering with the unconfined run-and-tumble pattern. We hypothesise that the presence of the trap walls coupled with the shear flow hinders the process of flagella unbundling and re-bundling. As a result, a trapped cell can spend extended periods of time with its flagella either unbundled or partially bundled, during which the bacterium cannot actively propel itself. In addition to this phenotype, we also observed extreme events where cells consistently remain in the low-motility state for the entire duration of the trapping event, like the cellular motion reported in Figure S3(d).

Molaei *et al*.[3] and Qu *et al*.[4] reported similar swimming behaviour and termed it a slow random walk. Bhattacharjee *et al*.[5] also observed a strikingly similar behaviour for *E. coli* trapped in cell-sized cavities within a porous matrix, whereby hindered bundling suppressed the swimming speed for tens of seconds. In addition, since flagella are typically much longer than the cell body, they are likely not to be located entirely within the trap (see Figure 1(a) in the main manuscript). Flagella are therefore likely to be exposed to higher flow rates and shear which may further contribute to impeded bundling.

In order to classify these swimming phenotypes, we use the asymmetry or skewness, s_{PDF}, of the single-cell motility distributions (as reported in the PDFs of Figure S3), to quantify the relative contribution of each motility state. Similarly, the fraction of trapping time spent by a cell in the low-motility state, τ_{low}, can be quantified by summing the time spent in the low motility state below a threshold motility. This threshold value is set to 5 here as indicated by the red lines in the second column of Figure S3. If a cell remained consistently in a certain motility state, it would produce a constant noise over time and therefore a roughly symmetric unimodal distribution with a small value of skewness, as illustrated in Figure S3(d). This is also the case for a cell that spends equal time in each state, such as Figure S3(b). In this case, τ_{low} would allow us to distinguish the two behaviours, as seen in the relative insets of Figures S3(b) and (d) (τ_{low} = 0.34 and 1, respectively). If a cell spends more time in the high-motility state, its distribution will have $s_{PDF} < 0$ and small τ_{low} due to the presence of a minor low-motility peak (see Figure S3(a)), while the opposite holds for a cell that spends more time in the low-motility regime (i.e., $s_{PDF} > 0$ and large τ_{low}, as in Figure S3(c)).

The use of the above quantities enabled us to unveil the reason behind the larger fraction of *S.* Typhimurium cells belonging to the low motility sub-population compared to *E. coli*. This difference is

revealed by the distribution of s_{PDF} for motile cells, as shown in Figure S3(e). These histograms show that single-cell motility PDFs of *E. coli* feature a lower average skewness compared to *S.* Typhimurium (-0.4 compared to 0.9, respectively), suggesting that the latter, on average, spend longer in the low-motility state. This is also confirmed by the distributions of τ_{low} (Figure S3(f)) which indicate *E. coli* cells spend on average 25% of their trapping time in the low-motility state, while for *S.* Typhimurium this fraction is 61%. We note the significant fraction of *S.* Typhimurium cells where $\tau_{\text{low}} > 0.9$ which indicates extreme cases where cells are consistently swimming slower.

We speculate that this difference between the two bacteria relates to the larger number of flagella and accompanying flagellar motors typically found on *S.* Typhimurium cells compared to *E. coli*[2]. For a cell to keep running, all flagellar motors must consistently rotate in a counter-clockwise (CCW) direction. The more flagellar motors present on a cell, the greater the probability that one of them will stochastically switch from CCW to clockwise (CW) rotation. It has been demonstrated that not every flagellar motor on a single cell must switch to CW rotation for a tumble to occur (i.e. one motor switching to CW rotation is sufficient)[2,6]. However, the magnitude of the direction change from run to run does correlate with the number of unbundled flagella (i.e. the number of motors undergoing CW rotation during a tumble). Hence, cells with more flagella are predicted to tumble more frequently, which agrees with the differences observed here.

Overall, this analysis shows that the bimodality does not only arise from different cells swimming at different speeds, but that it also results from single cells oscillating between two states of motility in the hydrodynamic trap. In addition, we observed some cells consistently swimming at a lower speed. This means that the observed heterogeneity of motility is present both at the population level and the single-cell level.

SI 3. *E. coli* MG1655 exposed to trimethoprim and ampicillin

SI 3.1. Trimethoprim preserves bi-modality and steadily decreases motility

Figure S4 shows results for wild-type *E. coli* MG1655 exposed to 10 μg mL^{-1} trimethoprim (a bacteriostatic antibiotic). We note that trimethoprim causes a slow decay in the normalised motility compared to kanamycin, likely because the inhibition of tetrahydrofolic acid (THF) biosynthesis induced by trimethoprim does not have a direct effect on the flagellar motor. The reason for the decreased motility is instead filamentation, namely elongation of the cell body without division, as illustrated in Figures

S4(e)-S4(g). In fact, elongation of the cell body is known to reduce the bacterial swimming velocity and to hamper their rotational diffusivity[7,8].

This mechanism is also confirmed by the behaviour of the motility distributions shown in Figures S4(b)-S4(d). The primary high-motility peak smoothly decreases in amplitude and shifts to lower average values over the course of the antibiotic action, while the secondary peak remains approximately constant. We also note that, unlike in the case of kanamycin, the final distribution is still clearly bimodal and neither of the peaks overlaps with the non-motile and dead distributions (green and dead curves in Figure S4(d), respectively). Figure S4(h) confirms that both sub-populations are still present as the two curves do not intersect, and two distinct motility peaks are measured until the end of the experiment. In addition, the low-motility population (orange curve) is not significantly affected so that the overall loss of motility is mainly attributed to the filamentation of the main sub-population.

Overall, these considerations show that all bacteria are still actively swimming by the end of the experiment, with the high-motility fraction moving with reduced efficiency because of filamentation. This observation agrees with trimethoprim having a bacteriostatic action, as it inhibits bacterial division and induces filamentation[9]. In addition, longer bacteria are known to change their swimming behaviour from the characteristic “run-and-tumble” modality to a “run-and-stop” pattern and to desynchronise flagellar rotation, which lead to less effective swimming strategies[7]. In contrast, the resistant strain maintained its wild-type morphology upon treatment with trimethoprim (data not shown) and did not show any significant decrease in motility (orange curve in Figure S4(a)).

SI 3.2. Ampicillin induces a two-phase reduction of motility

Treatment with 10 μg mL^{-1} ampicillin (a bactericidal antibiotic) caused only a temporary loss of motility in a resistant *E. coli* strain (orange curve in Figure S5(a)), an effect that was also observed in similar conditions by tethering ampicillin-resistant *E. coli* to an AFM cantilever[10]. In contrast, the decrease of motility was irreversible for the susceptible strain (blue curve in Figure S5(a)) and continued until most cells lysed. This reduction in observed motility can be explained by two mechanisms. Firstly, ampicillin is known to interfere with the transcription of genes required for flagella assembly and activation[11]. Secondly, the inhibition of new cell wall synthesis generates pressure across the membrane. The increased pressure triggers a two-step process which consists of cellular elongation followed by the formation of a bulge to partially relieve the membrane pressure[12,13] (see Figures S5(e)-S5(g)).

These morphological changes are reflected in the motility curve, where the decrease also follows a two-stage behaviour (blue curve in Figure S5(a)), unlike the sharp decay observed for kanamycin (see the main manuscript) and the slower trend induced by trimethoprim (see section SI 3.1 above). Firstly, up to ~1.3 h after exposure to ampicillin, the decrease in motility is limited to about 85% of the maximum value, which can be ascribed to the initial filamentation process, similar to the effect induced by trimethoprim. However, as the number of bulged bacteria increases, a more abrupt drop of motility to ~50% is observed, since bulged bacteria are severely impeded in their swimming action. Nearly total cell lysis occurred after about ~1.8 h of exposure to the antibiotic.

The two-phase dynamic is also revealed by the analysis of the motility distributions over time, as shown in Figures S5(b)-S5(d). In the absence of antibiotics, we observed the typical bimodal distribution. After 0.8 h of exposure, the filamentation process caused the position of the primary motility peak to decrease, while after 1.6 h, both motility peaks have shifted to smaller values. The further decrease in the average value for both peaks can be ascribed to the bulging of the cell envelope significantly hindering the swimming ability of bacteria from both sub-populations. In fact, the formation of the bulge was observed in both low- and high-motility bacteria.

Notably, the low-motility sub-population in Figure S5(d) peaks at motility values higher than the non-motile and dead populations (green and black Gaussian distributions, respectively), showing that bulged bacteria were still actively swimming until cell death occurred through rupture of the cell. The time-dependent changes in motility observed in Figures S5(b)-S5(d) are summarised in Figure S5(h). Similar to the case of kanamycin and trimethoprim, the high-motility sub-population is affected more significantly by ampicillin, while the low-motility bacteria only show a modest decrease in motility.

As opposed to the susceptible case, resistant bacteria did not show any significant morphological changes (data not shown) and did not lyse, hence confirming that the effect described here is indeed induced by bacterial susceptibility to ampicillin.

SI 3.3. Sub-population switch induced by different antibiotics

The 50% motility threshold criterion for defining the high- and low-motility sub-populations is here applied to the cases of wild-type *E. coli* MG1655 exposed to kanamycin, trimethoprim and ampicillin in order to investigate the change in the sub-populations induced by antibiotics with different modes of action. Recall that f_1 is calculated as the area below the Gaussian fits for motilities larger than 50% of the maximum motility in the absence of any antibiotics. This is indicated by the grey-shaded regions beneath

the Gaussian fits in all histograms throughout the manuscript and SI, and is shown in Figure S6 (i.e. horizontal grey continuous line). Also recall that we fitted $f_1(t)$ with the exponential-logistic model represented by equation (1) in the main manuscript. In the case of purely susceptible colonies, equation (1) reduces to a single exponential curve for kanamycin and trimethoprim:

$$f_1(f_0, A, t^*; t) = f_0 + Ae^{-\frac{t}{t^*}} \qquad \text{(eq. S2)}$$

The two-stage motility loss observed for ampicillin is also reflected in the population switching dynamics (yellow data points in Figure S5). Consequently, a double exponential fit (i.e. a sum of two terms in the form of equation S2), was found to follow the trend more reliably. The best fit curves are represented by the continuous lines in Figure S5 for the three antibiotics. The observed differences in the decay constants of these exponential curves confirm that our method can distinguish between antibiotics with different modes of action.

SI 4. Modelling the behaviour of motility and sub-populations over time

In the case of the mixed susceptible-resistant *E. coli* populations discussed in the main manuscript, we have also fitted the time-dependent changes in the sub-populations with an exponential-logistic model that we derive below.

Let $x_s(t)$ and $x_R(t)$ be the fraction of susceptible and resistant bacteria over time, respectively, and their corresponding motilities are $M_s(t)$ and $M_R(t)$. The average motility $M(t)$ of such a mixed population is the weighted average:

$$M(t) = x_s(t)M_s(t) + x_R(t)M_R(t) \qquad \text{(eq. S3)}$$

where the bacterial fractions are reported as a fraction of 1, so that $x_S(t) + x_R(t) = 1$ at all times. Let us now quantify each of the four terms of equation S3.

- For a purely susceptible population, as suggested by the experimental data, $M_S(t)$ can be modelled with an exponential of the form:

$$M_s(t) = a + ce^{-bt}$$

With boundary conditions:

$$\begin{cases} M_s(t=0) = a + c = 1 \\ M_s(t \to \infty) = a = M_{S\infty} \end{cases}$$

where we assumed that $M_S(t)$ is normalised to its maximum and that it saturates to $M_{S\infty}$ by the end of the experiment. Therefore:

$$M_s(t) = M_{s\infty} + (1 - M_{s\infty})e^{-bt}$$

- For a purely resistant population, the experimental data (black curve in Figure 4(e) in the main manuscript), show that $M_R(t)$ is constant over time:

$$M_R(t) = M_{R\infty}$$

- Let us assume that upon the action of a near-MIC concentration of kanamycin, the division of susceptible bacteria is immediately stopped, such that we can take $x_S(t)$ to be constant over time and equal to the initial fraction x_{S0}:

$$x_S(t) = x_{S_0}$$

- Let us also assume that, upon the action of kanamycin, resistant bacteria continue to divide following a logistic model of growth:

$$x_R(t) = \frac{A}{1 + e^{k(\lambda - t)}}$$

with boundary conditions:

$$\begin{cases} x_R(t=0) = \dfrac{1}{1 + e^{k\lambda}} = x_{R_0} \\ x_R(t \to \infty) = A = 1 \end{cases}$$

where we assume that at very long times the resistant sub-population takes over the susceptible portion, such that its weight will be equal to 1. Therefore:

$$x_R(t) = \frac{1}{1 + \left(\frac{1}{x_{R_0}} - 1\right)e^{-kt}}$$

By substituting these expressions in the general formula for *M(t)* we obtain:

$$\begin{cases} M(t) = x_R(t)M_{R_\infty} + \; x_S(t)\left[M_{S_\infty} + \left(1 - M_{S_\infty}\right)e^{-bt}\right] \\ x_R(t) = \dfrac{1}{1 + \left(\dfrac{1}{x_{R_0}} - 1\right)e^{-kt}} \\ x_R(t) \; + \; x_S(t) \; = \; 1 \end{cases}$$

In our experiments, x_{S0}, x_{R0}, $M_{S\infty}$ and $M_{R\infty}$ are given parameters. The initial fractions of susceptible and resistant bacteria are chosen when preparing the bacterial cultures while $M_{S\infty}$ and $M_{R\infty}$ are taken from the purely susceptible and purely resistant curves, respectively, where b and k are used as fitting parameters. The exact same model can be applied to the time evolution of the fraction of the high-motility sub-population $f_1(t)$, whereby $M(t)$, $M_{S\infty}$ $M_{R\infty}$ can simply be substituted with the corresponding $f_1(t)$, $f_{1,S\infty}$ and $f_{1,R\infty}$. In the case of an unknown mixed population, x_{s0} and x_{r0} can also be used as fitting parameters, therefore enabling the quantification of heteroresistance in an uncharacterised bacterial sample.

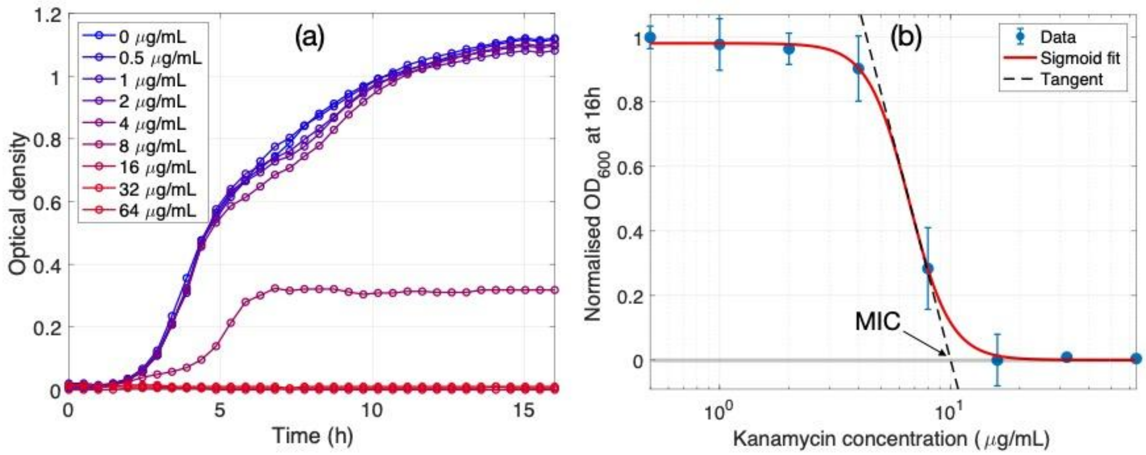

Fig. S1. (a) Optical density (OD) measured over time for *E. coli* MG1655 incubated with increasing concentrations of kanamycin. *E. coli* were suspended in a standard 96-well plate and the OD was measured at 600 nm with a microplate reader. (b) Normalised values of the OD (blue circles) at 16 h of incubation as a function of the concentration of kanamycin. Error bars denote standard deviation based on three biological replicates. Each biological replicate includes duplicate technical replicates. The continuous line is a best-fit curve to a sigmoidal function (eq. S1). The dashed line is the tangent to the sigmoidal function at its inflection point and is used to quantify the MIC, as indicated by the black arrow.

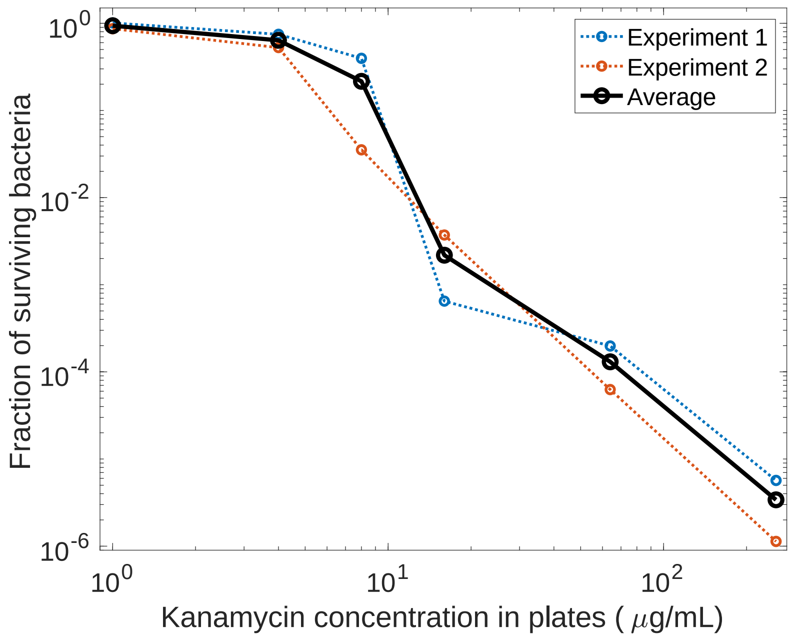

Fig. S2. Results of the population analysis profile (PAP) conducted with *S*. Typhimurium LT2 on kanamycin LB agar plates. Blue and orange lines refer to two independent replicates, each consisting of two plates per concentration. The black line represents the average between the two replicates and shows how killing spans over a >8-fold concentration of drug and that it is bi-phasic, indicating heteroresistance.

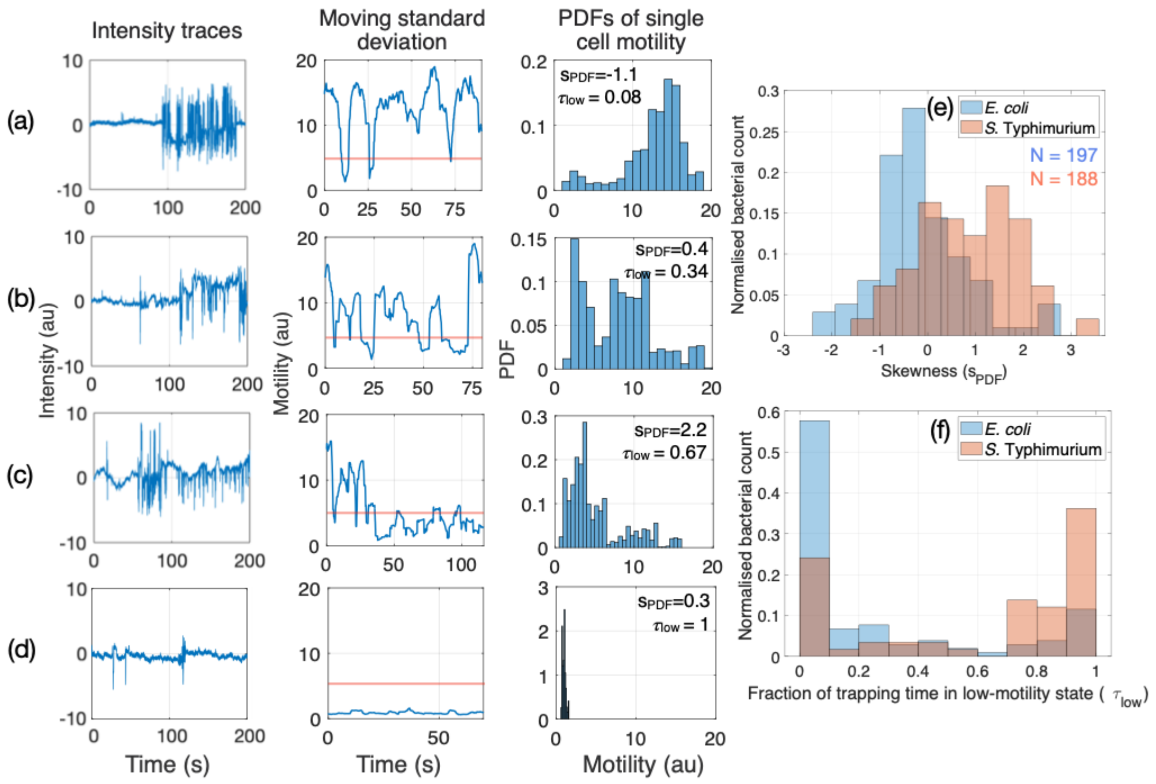

Fig. S3. Analysis of time-dependent single-cell motility reveals different swimming phenotypes. Columns (left to right) show real-time intensity traces, moving standard deviation of intensity signal, and probability density function (PDF) for single-cell trapping events, respectively. (a) A typical cell that spends more time running than tumbling inside the trap produces a single-cell motility PDF which is negatively skewed (s_{PDF} = -1.1). (b) The single-cell motility PDF has a skewness closer to zero (s_{PDF} = 0.4) when runs and tumbles occur with the same frequency. (c) A typical cell spending more time tumbling rather than running yields a PDF with a positive skewness (s_{PDF} = 2.2). (d) A cell moving much slower than the rest of the population which is undergoing a slow random walk (no running or tumbling) also produces a PDF with low skewness (s_{PDF} = 0.3). (e) Distributions of the skewness of the single-cell PDFs for motile *E. coli* and *S*. Typhimurium. (f) Distribution of the fraction of time spent in the low-motility state (τ_{low}) by trapped *E. coli* and *S*. Typhimurium reveals that, on average, *S*. Typhimurium spend longer in the low-motility state compared to *E. coli*.

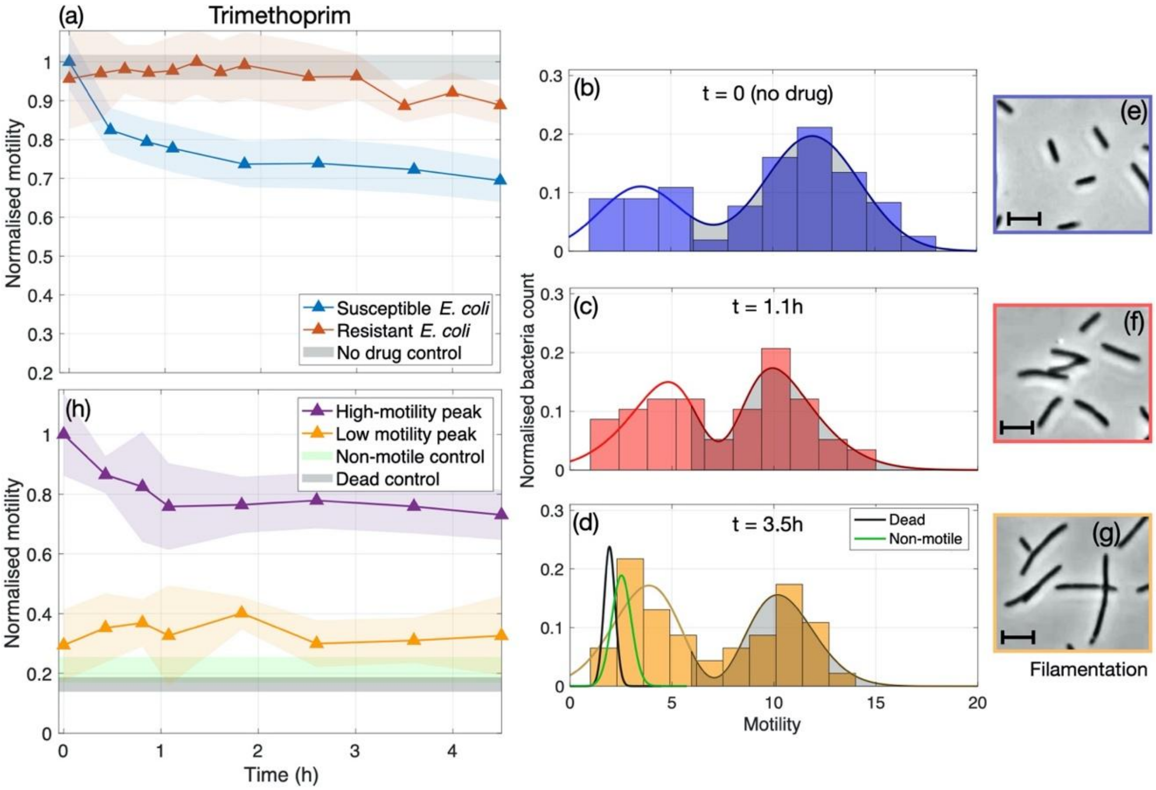

Fig. S4. *E. coli* MG1655 exposed to 10 µg/mL trimethoprim. (a) Average motility values over time measured for susceptible (blue curve) and trimethoprim-resistant (orange curve) *E. coli* MG1655 exposed to 10 µg/mL of trimethoprim (a bacteriostatic antibiotic). The grey-shaded area refers to a separate no-drug control as before. (b, c, d) Histograms of motility values before treatment (blue), and after 1.1 h (red) and 3.5 h (yellow) of exposure to trimethoprim. Continuous lines represent bi-modal Gaussian fits of the histogram counts. The grey-shaded regions beneath the Gaussian fits in all histograms represents a 50% motility threshold defining two sub-populations of different motility (i.e., low- and high-motility, respectively). (e, f, g) Phase-contrast microscopy images showing typical bacterial morphology at different time points, clearly illustrating the filamentation induced by trimethoprim. Scale bar is 2 µm. (h) Average motility value for each Gaussian fit component over time. Error bars denote standard deviation based on two biological replicates.

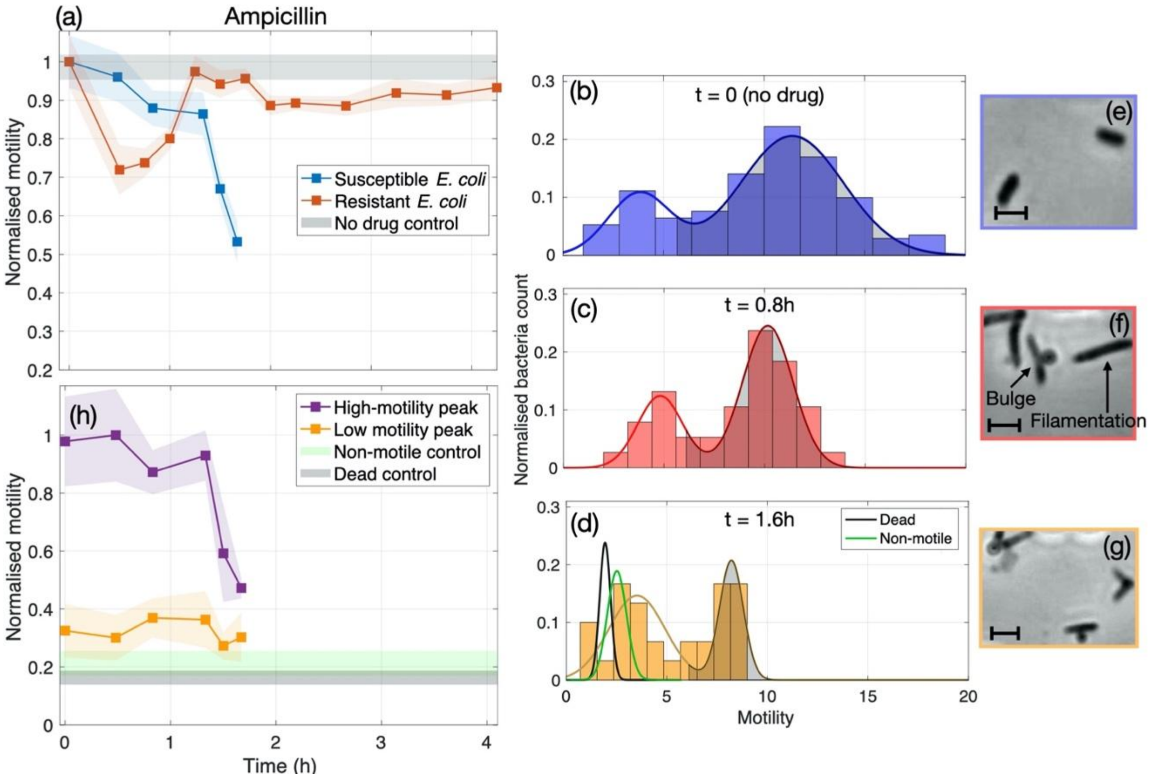

Fig. S5. *E. coli* MG1655 exposed to 10 μg/mL ampicillin. (a) Average motility values over time measured for ampicillin-resistant (orange curve) and ampicillin-susceptible (blue curve) *E. coli* MG1655 exposed to 10 μg/ml of ampicillin (a bactericidal antibiotic). The grey-shaded area refers to a no-drug control, where bacteria were not exposed to ampicillin at any point. (b, c, d) Histograms of motility values before (blue), after 0.8 hours (red) and after 1.6 hours (yellow) of exposure to ampicillin, respectively. Continuous lines represent bi-modal Gaussian fits of the histogram counts. Motility distributions for non-motile (green) and dead bacteria (black) are provided for comparison. The grey-shaded regions beneath the Gaussian fits in all histograms represents a 50% motility threshold defining two sub-populations of different motility (i.e. low- and high-motility, respectively). (e, f, g) Phase-contrast microscopy images showing typical bacterial morphology at different time points, illustrating the early filamentation phase followed by the formation of the cell envelope bulge induced by ampicillin. Scale bar is 2 μm. (h) Peak values of each Gaussian fit component over time. Nearly all susceptible *E. coli* cells had lysed after ~1.8 h of exposure to ampicillin. Error bars denote standard deviation based on two biological replicates.

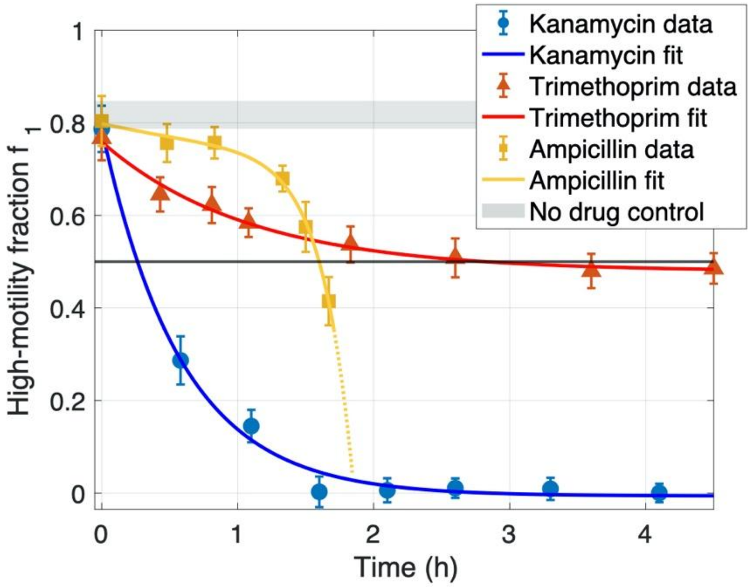

Fig. S6. Fraction of high-motility (f_1) bacteria over time upon the exposure of *E. coli* MG1655 to 10 µg/mL kanamycin (blue circles), trimethoprim (red triangles) and ampicillin (yellow squares). Continuous lines are exponential fits to the kanamycin and trimethoprim data (blue and red, respectively) and a double exponential fit to the ampicillin data (yellow). The horizontal grey line denotes a threshold of 50% of the maximum motility in the absence of any antibiotics. Error bars denote the standard deviation of f_1 based on three biological replicates for kanamycin and two for ampicillin and trimethoprim.

SI References

1. Darnton, N. C., Turner, L., Rojevsky, S. & Berg, H. C. On torque and tumbling in swimming Escherichia coli. *J. Bacteriol.* **189**, 1756–1764 (2007).
2. Turner, L., Ryu, W. S. & Berg, H. C. Real-time imaging of fluorescent flagellar filaments. *J. Bacteriol.* **182**, 2793–2801 (2000).
3. Molaei, M., Barry, M., Stocker, R. & Sheng, J. Failed escape: Solid surfaces prevent tumbling of Escherichia coli. *Phys. Rev. Lett.* **113**, 1–6 (2014).
4. Qu, Z., Temel, F. Z., Henderikx, R. & Breuer, K. S. Changes in the flagellar bundling time account for variations in swimming behavior of flagellated bacteria in viscous media. *Proc. Natl. Acad. Sci. U. S. A.* **115**, 1707–1712 (2018).
5. Bhattacharjee, T. & Datta, S. S. Bacterial hopping and trapping in porous media. *Nat. Commun.* **10**, 2–10 (2019).
6. Mears, P. J., Koirala, S., Rao, C. V., Golding, I. & Chemla, Y. R. Escherichia coli swimming is robust against variations in flagellar number. *Elife* **2014**, 1–18 (2014).
7. Maki, N., Gestwicki, J. E., Lake, E. M., Kiessling, L. L. & Adler, J. Motility and chemotaxis of filamentous cells of Escherichia coli. *J. Bacteriol.* **182**, 4337–4342 (2000).
8. Guadayol, Ò., Thornton, K. L. & Humphries, S. Cell morphology governs directional control in swimming bacteria. *Sci. Rep.* **7**, 1–13 (2017).
9. Cushnie, T. P. T., O’Driscoll, N. H. & Lamb, A. J. Morphological and ultrastructural changes in bacterial cells as an indicator of antibacterial mechanism of action. *Cell. Mol. Life Sci.* **73**, 4471–4492 (2016).
10. Longo, G. *et al.* Rapid detection of bacterial resistance to antibiotics using AFM cantilevers as nanomechanical sensors. *Nat. Nanotechnol.* **8**, 522–526 (2013).
11. Kaldalu, N., Mei, R. & Lewis, K. Killing by Ampicillin and Ofloxacin Induces Overlapping Changes in Escherichia coli Transcription Profile. *Antimicrob. Agents Chemother.* **48**, 890–896 (2004).
12. Yao, Z., Kahne, D. & Kishony, R. Distinct Single-Cell Morphological Dynamics under Beta-Lactam Antibiotics. *Mol. Cell* **48**, 705–712 (2012).
13. Huang, K. C., Mukhopadhyay, R., Wen, B., Gitai, Z. & Wingreen, N. S. Cell shape and cell-wall organization in Gram-negative bacteria. *Proc. Natl. Acad. Sci. U. S. A.* **105**, 19282–19287 (2008).
